# World Salt Awareness Week: A call to action for reducing salt intake in Malaysia

**DOI:** 10.51866/cm.929

**Published:** 2025-05-27

**Authors:** Yook Chin Chia, Siew Mooi Ching, Nik Sherina Hanafi

**Affiliations:** 1 MBBS, MRCP, FRCP, Department of Clinical Medicine and Surgery, Faculty of Medicine and Life Sciences, Sunway University, Bandar Sunway, Selangor, Malaysia.; 2 Department of Primary Care Medicine, Faculty of Medicine, Universiti Malaya, Lembah Pantai, Kuala Lumpur, Malaysia.; 3 Ageing, Health and Wellbeing Research Centre, Faculty of Medicine and Life Sciences, Sunway University, Bandar Sunway, Selangor, Malaysia. Email: ycchia@sunway.edu.my; 4 MD, MMed (Family Medicine), PhD, Department of Family Medicine, Faculty of Medicine and Health Sciences, Universiti Putra Malaysia, Serdang, Selangor, Malaysia.; 5 Malaysian Research Institute on Ageing (MyAgeing), Universiti Putra Malaysia, Serdang, Malaysia.; 6 Research Centre of Excellence Nutrition and Non-communicable Diseases, Faculty of Medicine and Health Sciences, UPM, Malaysia.; 7 MBBS, MMed (Fam Med), PhD, Department of Primary Care Medicine, Faculty of Medicine, Universiti Malaya, Lembah Pantai, 50603 Kuala Lumpur, Malaysia.

**Keywords:** World, Salt, Awareness, Malaysia

## Abstract

Cardiovascular diseases remain a primary contributor to death worldwide, with hypertension being a key determinant. Excessive salt intake is a contributing factor of high blood pressure and cardiovascular diseases. To address this, the World Health Organization recommends keeping daily salt consumption under 5 g and aiming for a 30% decrease by 2025. In Malaysia, efforts to reach this target have faced delays. A local study found that 79% of Malaysians consume an average of 7.9 g of salt per day, which is significantly higher than the WHO’s recommendations. Despite efforts such as voluntary food reformulation, mandatory sodium labelling and public education campaigns, challenges remain. Industry reluctance, low consumer awareness and inadequate enforcement slow down such efforts. This commentary reviews these issues and suggests applicable approaches to strengthen Malaysia’s salt reduction strategies.

## Introduction

Cardiovascular diseases (CVDs) remain a common cause of death globally, with hypertension as a significant contributors towards its development.^1^ According to the Global Burden of Disease Study, high salt consumption has been linked with approximately 1.89 million deaths per year.^2^ Thus, reducing salt intake is among the efficient strategies to prevent CVDs.^3^ To address this issue, the World Health Organization (WHO) recommends a daily dietary salt intake of less than 5 g and has set an international goal of reducing salt intake by 30% by 2025. To date, the progress to achieve this target is rather slow despite various countries’ efforts of implementing campaigns for salt reduction.

However, a few countries have been quite successful in reducing population salt intake. One of such country is the United Kingdom with its’ drive to reduce salt content in foods. The UK’s salt reduction programme is often cited as successful and cost-effective, as it managed to achieve a 15% reduction in salt intake by decreasing sodium content in processed foods from 2003 to 2011. This level of reduction was associated with a decrease in blood pressure and CVD-related deaths in the population.^3^ Similarly, South Africa showed positive outcomes, with a decrease of 1.15 g per day in sodium intake within 2 years of introducing mandatory sodium limits in processed foods.^4^ These examples demonstrate the importance of structured and enforced policies in reducing population-wide sodium consumption. Such enforced policies in salt reduction could benefit countries including Malaysia, a nation with a generally high sodium intake.

**Current salt reduction strategies in Malaysia**

The salt reduction strategy for Malaysia started from 2015 to 2020^5^, and was subsequently extended to 2021-2025.^6^ The Malaysian Salt Reduction Strategy (2021-2025) emphasises monitoring, awareness and food modification to lower salt consumption. Monitoring efforts include measuring sodium concentrations using 24-h urinary sodium analysis, focusing not only on processed foods and home-cooked meals but also on foods from street vendors, restaurants, school canteens and food courts.^7^

Awareness campaigns focusing on school children, economically disadvantaged populations and patients with chronic conditions have been conducted while supporting label reading and reduced-salt options, with support from nongovernment organisation (NGO).

In food modification, 62 food manufacturers are recommended to voluntarily begin reducing sodium, with compulsory salt content disclosure from January 2024, which indicates a positive progress.

**Challenges and policy gaps**

In Malaysia, high salt intake continues to be a concern. The Malaysian Community Salt Survey (MyCoSS) reported 79% of Malaysians consume an average of 7.9 g of salt daily, which is 2.9 g higher than the WHO’s recommended level.^8^ Most Malaysians assume low-salt diets are tasteless and are unaware that eating fried vegetables from Chinese restaurants, consuming bread daily and cooking food with soy sauce all contribute to excessive hidden salt intake.^9^ High-salt foods such as instant noodles, street foods and condiments remain popular due to convenience and delicious taste; furthermore, salt disclosures are lacking in the country.^10,11^

Food reformulation requires additional cost. This will be a challenge for small and medium enterprises to align with the sodium reduction policy, to maintain the product taste and to balance the cost. Without mandatory salt restriction, the impact of initiatives is uncertain.

The WHO’s Sodium Country Score Card ranks Malaysia at Level 4, indicating a strong policy commitment in salt reduction initiatives,^12^ but enforcement is lacking. There is a lack of monitoring of salt content in commercialised foods, and no initiatives have been introduced to tax high-salt diets in Malaysia.

**Strengthening Malaysia’s salt reduction efforts**

A focused, well-structured and ongoing approach is needed, especially for high-salt intake countries such as Malaysia. A multidisciplinary approach involving mandatory regulations on salt reduction across the entire food industry, increased consumer awareness and stronger industry engagement must be expanded to achieve success similar to that seen in the UK and South Africa.

First, strengthening mandatory policies is important by setting maximum sodium thresholds for processed foods based on the WHO’s recommendations. Second, clearer dietary information should be provided, and the public should be educated on how to read food labels before purchasing. Third, taxes should be introduced on high-sodium products to help control salt consumption while generating funding for public health campaigns.

Enhancing public education is another measure. In conjunction with World Salt Awareness Week, every effort must be made to raise awareness towards the benefits of salt reduction and to promote healthier habits by highlighting the hidden salt content in daily diets.

Policymakers could partner with community programmes such as Kafeteria Sihat, Kelab Doktor Muda and Program Siswa Sihat to encourage better dietary habits in schools and workplaces.^13^ There are efforts to increase collaboration with NGOs such as the Malaysian Society for World Action on Salt, Sugar and Health (MyWASSH) in engaging remote and at-risk groups. MyWASSH,^14^ a newly formed society in 2021, consists of family medicine specialists and allied healthcare professionals, including dietitians. It collaborates with the Malaysian Society of Hypertension to educate the public on the dangers of excessive salt intake and to promote healthier dietary habits.^15^

Industry collaboration is also important, as technical support can be provided to manufacturers to enable the progressive reduction of salt intake and the development of alternative formulations. The implementation of these changes takes time and is executed in phases to buffer potential resistance from the industry. Companies’ fears, that their sales may be negatively affected when they reduce the salt content in their foods should be acknowledged, with reassurance that this may not always be the case. Other potential challenges related to implementation or stakeholder buy-in include the standardisation of salt labelling in terms of the unit used, such as gram or percentage, and the cut-off point for the maximum amount of salt allowed as well as industry agreement to update the latest food labelling in digital databases. Another measure that could be taken is to expand the Malaysian Food Composition Database to enable consumers to easily find low-salt products available in the country.

Malaysia should learn from the UK and South Africa’s success in salt reduction through mandatory salt restriction. Having an Asia Salt Consortium and engaging in ASEAN food policy discussions can promote standardisation in salt labelling and encourage the reformulation of foods by the food industry to support salt reduction.

Regular monitoring through urinary sodium assessments and dietary surveys is crucial to evaluate the effectiveness of implemented measures and to guide policy adjustments. Digital tools including mobile applications can be used to improve real-time data tracking of daily food consumption using food logs or barcode scanning. Salt metres can be used to check the salt level of home-cooked food based on the traffic light colour. Dietary salt intake could be linked to web-based education modules that teach individuals how to identify hidden salt and outline actions to take such as how much salt should be consumed when home-cooked meals fall under high-salt categories. Additionally, electronic health records uploaded to a website can be accessed by healthcare providers during routine clinic visits for further intervention, as shown in a study conducted in Japan.^16^ Although there was a significant difference between the intervention and control groups, a local study is still necessary, as cultural factors and health literacy related to digital use may differ in this country.

Healthcare integration by incorporating salt intake assessment in regular medical evaluations and providing counselling sessions on salt reduction by trained healthcare providers are other positive venture. Primary care physicians play an important role in this salt reduction programme, as they are the front lines for patients with non-communicable diseases, especially hypertension. Thus, it is important to have salt intake assessment during daily clinical practice. Community screening programmes should also evaluate sodium-related health risks, such as hypertension, to carry out preventive actions.

## Conclusion

Every effort involving policymakers, healthcare providers, the public and industry partners is needed to achieve the recommended salt intake target of less than 5 g in Malaysia. World Salt Awareness Week provides an opportunity to increase awareness towards low-salt diets and to identify daily foods with hidden salt content. A call to action for reducing salt intake in Malaysia by introducing a mandatory law on salt restriction is urgently needed.
